# Polysaccharides and Polyacrylamide as Linear Polymeric Stabilizers for Zwitterionic Short-Chain Fluorocarbon Surfactant: Interfacial Properties, Apparent Viscosity, and Foam Performance

**DOI:** 10.3390/polym17233112

**Published:** 2025-11-24

**Authors:** Wenjun Zhao, Ziyang Zhu, Zhisheng Xu, Long Yan

**Affiliations:** 1 Institute of Disaster Prevention Science and Safety Technology, School of Civil Engineering, Central South University, Changsha 410075, China; 2 State Key Laboratory of Fire Science, University of Science and Technology of China, Hefei 230026, China

**Keywords:** foam drainage, foam evolution, polymeric stabilizer, short-chain fluorocarbon surfactant, surface and interfacial activity, apparent viscosity

## Abstract

Polymeric stabilizers play a critical role in enhancing the stability and performance of firefighting foams. This study evaluated the influence of three polymeric stabilizers (xanthan gum, XG; polyacrylamide, PAM; sodium carboxymethyl cellulose, CMC-Na) on the performance of foam solutions formulated with a zwitterionic short-chain fluorocarbon surfactant. The investigation focused on three performances: interfacial properties, apparent viscosity (at a fixed rotational speed), and foam performance, employing interfacial tension analysis, viscosity measurement, dynamic foam analysis, and foam drainage testing. Results indicate that XG and CMC-Na slightly decrease interfacial activities, reducing spreading coefficients 6.34–15.78% and 0.68–6.35%, respectively. However, these polymeric stabilizers substantially increase apparent viscosity through hydrogen bond network formation, which effectively mitigates foam coarsening and drainage. When adding 0.10 wt.% XG, the foam solution exhibits a characteristic coarsening time of 724.64 s and a 25% drainage time of 1519.15 s. Conversely, PAM exhibits a concentration-dependent dual effect. When below 0.06 wt.%, PAM enhances interfacial properties and foam stability. However, at elevated concentrations, excessive PAM aggregates at interfaces and forms entangled networks that inhibit surfactant adsorption. This impairs foam formation and accelerates foam structural evolution, increasing variation in bubble size and promoting foam drainage by 8.63–57.88%. These findings provide crucial reference for applying polymeric stabilizers in short-chain fluorocarbon surfactant systems.

## 1. Introduction

Aqueous film forming foam (AFFF) has been widely recognized as the one of the most efficient extinguishing agents for hydrocarbon fuel fires [1]. During fire suppression, AFFF produces substantial and highly stable foam that effectively blankets the surface of burning hydrocarbon fuels. AFFF achieves rapid fire suppression through cooling, isolating and smothering effects, facilitated by a stable foam blanket and a rapidly spreading aqueous film [2,3]. The exceptional performance of AFFF primarily stems from the complementary roles of fluorocarbon surfactants and foam stabilizers, which are the critical functional components of AFFF.

Fluorocarbon surfactants have attracted considerable research attention due to their distinctive hydrophobicity and oleophobicity. These compounds demonstrate outstanding surface activity, which directly affects the foaming and spreading performance of firefighting foams [4]. However, growing environmental concerns have revealed that perfluoroalkyl and polyfluoroalkyl substances (PFAS) exhibit environmental persistence, posing great ecological hazards and threatening sustainable development. Regulatory agencies have imposed strict bans on long-chain PFAS like perfluorooctane sulfonic acid, perfluorooctanoic acid, and their derivatives, significantly impacting firefighting operations [5,6]. This has motivated the urgent development of eco-friendly foam extinguishing agents suitable for hydrocarbon fires. Current research focuses on two primary alternatives: fluorine-free foams and environmentally friendly fluorocarbon surfactant-based foams [7,8,9]. Fluorine-free foams, typically based on silicone and hydrocarbon surfactants, exhibit reduced environmental persistence [10]. However, the absence of C–F bonds compromises oleophobicity, impairing spreading and sealing capabilities on hydrocarbon fuel surfaces [11]. Therefore, fluorine-free foams require longer application time and higher consumption to achieve fire suppression efficiency comparable to traditional AFFFs, resulting in increased operational costs and greater biodegradation burdens [8,11]. In contrast, environmentally friendly fluorocarbon surfactant-based foams maintain considerable surface activity and oleophobicity, while reducing environmental impacts by appropriately reducing F content [12,13]. In our previous work, a betaine-type zwitterionic short-chain fluorocarbon surfactant (PFH-BZ) was synthesized. The results revealed that PFH-BZ exhibited excellent surface activity, with a critical micelle concentration (*cmc*) of 1.05 mmol/L and a surface tension at *cmc* (*γ_cmc_*) of 18.45 mN/m [14]. Especially, when compounded with *N*,*N*-dimethyldodecan-1-amine oxide (OB-2) at a 1:2 molar ratio, the system achieved a *cmc* of 0.14 mmol/L and a *γ_cmc_* value of 22.13 mN/m. This presents a feasible pathway for developing efficient and environmentally friendly foam extinguishing agent.

However, as a thermodynamically unstable structure, surfactant-stabilized foam demonstrates limited stability [15]. Therefore, foam stabilizers are important for retarding foam collapse and improving extinguishing performance. Beyond surfactants themselves, various types of foam stabilizers have been explored to enhance foam stability. For instance, our previous works have examined the influence of inorganic salts and long-chain alcohols on foam solutions formulated with a short-chain fluorocarbon surfactant [14,16]. These findings indicate that inorganic salts moderately improved foam stability via electrostatic shielding, while long-chain alcohols retarded foam structural evolution through interfacial adsorption and participation in micellization. Diethylene glycol butyl ether (DGBE), commonly employed as a solvent, has been shown to impact the micellization and surface adsorption of surfactants [4,17]. This resulted in reduced surface tension and promoted the formation of wetter and smaller bubbles, which in turn improved foam spreadability, stability, and sealing capacity. Although these additives could provide stabilization effects, their performance still fell to meet the standards (such as ISO 7203-2019 and GB 15308-2025) and firefighting requirements [18,19]. Recently, nanoparticles (NP) have gained widespread attention as foam stabilizers. By forming flocs or jamming the Plateau borders, NPs constructed physical barriers between bubbles and increased solution viscosity, ultimately leading to highly stable foams [20,21]. However, this excellent stabilization effect is sensitive to NP concentration. At low concentrations, NPs accelerated foam structural evolution, while high concentrations would lead to over-aggregation, both adversely impairing foam stability [20,22].

Given the limitations of above components, polymers have emerged as promising foam stabilizers due to their unique macromolecular structure and excellent viscosity-increasing effects. Currently, these polymeric stabilizers are widely applied in diverse fields, including food processing, cosmetics formulation, and oil recovery [23,24,25]. In fire extinguishing applications, polysaccharide polymers have attracted wide attention owing to their biocompatibility and excellent stabilization effect. Several studies have systematically investigated the effects of xanthan gum (XG), guar gum, and sodium carboxymethyl cellulose (CMC-Na) on silicone surfactant systems [26,27,28,29]. The results revealed that these polymers not only enhanced surface activity, but also significantly improved foam stability via retarding foam drainage and coarsening processes. Particularly, XG facilitated the formation of a protective gel film at the foam-fuel interface, effectively mitigating the destructive impact of polar fuels on foam structure [30]. Through synergistic interactions between XG, gelatin, and MgCl_2_, an environmentally friendly foam extinguishing agent was successfully developed, with an extinction time of 40.95 s and a burnback time of 614 s in diesel fire [31]. Beyond polysaccharide polymers, linear polymers, such as polyacrylamide (PAM), have also been utilized to improve foam stability and oil recovery in petroleum industry [32]. In addition to its viscosity-increasing effect, the experimental results indicates that amphiphilic feature of PAM facilitated the formation of complexes with sodium dodecylbenzene sulfonate at the gas–liquid interface, thereby increasing repulsion between thin films [33]. This effectively enhanced foam film strength and stability. Furthermore, simulation results reveal that as PAM concentration increases, water molecules around the hydrophilic groups of sodium dodecyl sulfate gradually increased, enhancing the water-holding ability and ultimately retarding foam drainage [34]. Despite these advances, current research on polymeric stabilizers primarily focuses on silicone and hydrocarbon surfactants. This leaves critical knowledge gaps regarding their interactions with short-chain fluorocarbon surfactants. Moreover, existing studies exhibit a notable lack of systematic investigations into interfacial phenomena and microscopic foam stabilization mechanisms, as the majority of research has primarily focused on characterizing macroscopic foam stabilization properties of polymeric stabilizers.

In this study, XG, CMC-Na, and PAM were employed as foam stabilizers to investigate their effects on the performance of foam solutions formulated with a zwitterionic short-chain fluorocarbon surfactants. Through comprehensive analysis of surface tension, interfacial tension, apparent viscosity (at a fixed rotational speed), foam microscopic structural evolution, and foam drainage property, a possible governing mechanism was further established. This study not only advances the fundamental understanding of polymer-stabilized short-chain fluorocarbon surfactant-based foam systems, but also provides a theoretical basis and experimental support for developing highly efficient and environmentally friendly foam extinguishing agents.

## 2. Materials and Methods

### 2.1. Materials

The synthetic route of zwitterionic short-chain fluorocarbon surfactant (PFH-BZ) is provided by our previous work [14]. *N*,*N*-dimethyldodecan-1-amine oxide (OB-2, 30%) was obtained from Lvsen Chemical Co., Ltd. (Linyi, China). Sodium hydroxide (NaOH, ≥96.0%) was obtained from Xilong Scientific Co., Ltd. (Shantou, China). Xanthan gum (XG, USP grade), urea (≥99.0%), ethylene glycol (≥98.0%), 2-(2-butoxyethoxy)ethanol (CP grade), and cyclohexane (≥99.7%) were purchased from Macklin Biochemical Technology Co., Ltd. (Shanghai, China). Sodium carboxymethyl cellulose (CMC-Na, ≥99.0%, apparent viscosity: 1200–1400 mPa·s) was supplied by Haohong Biomedical Technology Co., Ltd. (Shanghai, China). Polyacrylamide (PAM, molecular weight: 25 million) was bought form Daqian Environmental Protection Technology Co., Ltd. (Foshan, China). 0# diesel oil was provided by China Petroleum & Chemical Co., Ltd. (Beijing, China).

### 2.2. Preparation of Foam Solution

The foam solution was prepared by combining additive concentrate and surfactant concentrate. The additive concentrate was composed of polymeric stabilizer, urea, ethylene glycol, and 2-(2-butoxyethoxy)ethanol. Concurrently, the surfactant concentrate was prepared using a PFH-BZ/OB-2 compounding system at a molar ratio of 1:2. The pH value of the surfactant solution was adjusted to 7–8 by adding NaOH aqueous solution. The two concentrated solutions were then mixed under continuous mechanical stirring. The resulting mixture was subsequently diluted with deionized water to obtain the final foam solution. The concentrations of these key components of final foam solution are summarized in Table 1. In this study, XG, PAM, and CMC-Na were selected as the polymeric stabilizers, and their concentrations were varied as indicated in Table 1.

### 2.3. Measurements and Characterization

#### 2.3.1. Surface and Interfacial Activities Test

The surface tension and interfacial tension of foam solutions were measured using a JYW-200A automatic surface and interfacial tensiometer (Chengde Dingsheng Testing Machine Testing Equipment Co. Ltd., Chengde, China), employing the Du Nouy ring method. Each measurement was repeated three times, and the average value was reported as the test result. The spreading coefficient (*S*) was determined based on Equation (1):

(1) $$S=\gamma_{o}-\gamma_{w}-\gamma_{o-w}$$

where *S* denotes the spreading coefficient at oil-water interface, *γ_o_* is the surface tension of oil phase (27.27 ± 0.03 mN/m for 0# diesel oil and 24.57 ± 0.02 mN/m for cyclohexane), *γ_w_* is the surface tension of foam solution, *γ_o−w_* indicates the interfacial tension between oil phase (diesel oil or cyclohexane) and foam solution.

#### 2.3.2. Apparent Viscosity Test

The apparent viscosity of foam solutions was measured by an NDJ-5S digital rotational viscometer (Shanghai Lichen-BX Instrument Technology Co., Ltd., Shanghai, China). To ensure accuracy, the measurement was conducted using a 0# rotor at a fixed and optimized rotational speed of 60 rpm (full scale of 20 mPa·s) or 30 rpm (full scale of 20 mPa·s). Each measurement was repeated five times, and the average value was reported as the test result.

#### 2.3.3. Foam Morphology Evolution Test

The foam morphology was captured using a DFA100 dynamic foam analyzer (Kruss Scientific Instruments Co., Ltd., Hamburg, Germany). In each measurement, 20 mL foam solution was injected into the glass columns via syringe. Subsequently, aeration was supplied at a controlled flow rate of 0.2 L/min for 45 s. The foam morphology at a height of 65 mm was recorded with a resolution of 2 fps and a field of view area of 7.89 × 6.67 mm^2^. The foam column height and bubble diameter were accessed using the accompanied analysis software.

#### 2.3.4. Foam Drainage Property Test

The foam drainage property was characterized using a double-syringe foaming device and a drainage measurement apparatus. Prior to each measurement, both the foam solution and the drainage apparatus were thermally equilibrated at 50 °C by a circulating water bath. For each measurement, 20 mL foam solution and 40 mL air were homogeneously foamed using the double-syringe device. The resultant foam was immediately transferred to the drainage apparatus, where the drainage mass was continuously recorded. The time required to drain 25% of the initial foam mass was recorded as the 25% drainage time (*t*_25%_). Each measurement was repeated three times, and the average value was reported as the test result.

## 3. Results and Discussion

### 3.1. Effect of Polymeric Stabilizers on the Interfacial Properties of Foam Solution

**Figure 1:**
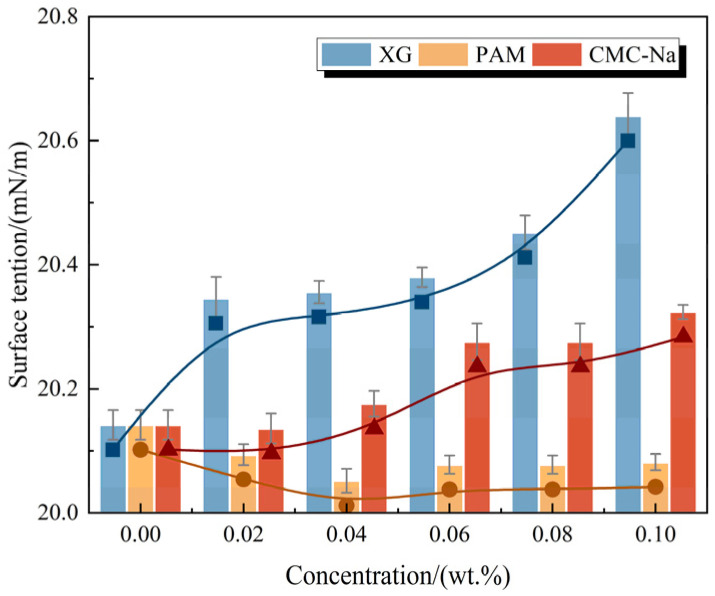
Surface tension of foam solutions containing polymeric stabilizers.

The surface tensions of foam solutions containing polymeric stabilizers are presented in Figure 1. The original foam solution exhibits high surface activity, as evidenced by a surface tension of 20.14 mN/m. As illustrated in Figure 1, polymer concentration exhibits a minor effect on the surface activity of the foam solution. Specifically, adding XG and CMC-Na slightly increases the surface tension to 20.43 ± 0.11 mN/m and 20.24 ± 0.01 mN/m, respectively. In contrast, the introduction of PAM reduces the surface tension to 20.08 ± 0.01 mN/m, representing a 0.30% decrease. These discrepancies can be attributed to the distinct interactions between the polymers and surfactant molecules at the surface. As highly hydrophilic macromolecular polysaccharide polymers, XG and CMC-Na competitively interfere with surfactant adsorption at the interface through steric hindrance effects, thereby slightly reducing the surface activity. Similar behavior is reported of various researchers while studying the interaction between these polymers and different surfactants [28,35,36,37]. In contrast, as a linear polymer with lower hydrophilicity, PAM demonstrates enhanced surface adsorption propensity [33,38]. In addition, PAM promotes the surface adsorption of surfactants, leading to an efficient surface tension reduction. However, it is noteworthy that these surface tension values do not exhibit a gradual decrease trend with increasing PAM concentration. This observation indicates that the interaction between PAM and surfactants fails to satisfy the stringent criteria for synergistic behavior, where the combined effect quantitatively exceeds the sum of individual contributions.

**Figure 2:**
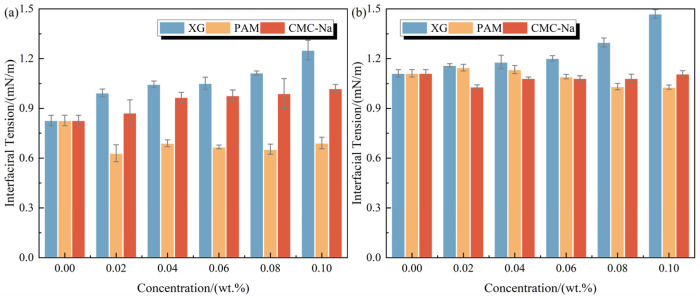
Interfacial tension between oil and foam solutions containing polymeric stabilizers: (**a**) diesel and (**b**) cyclohexane.

The interfacial tension between oil (0# diesel oil or cyclohexane) and foam solutions containing polymeric stabilizers is displayed in Figure 2, with the corresponding spreading coefficients (*S_DO_* for diesel oil and *S_CYH_* for cyclohexane) calculated in Table 2. The original foam solution exhibits high interfacial activity, as evidenced by interfacial tensions of 0.83 mN/m with diesel and 1.11 mN/m with cyclohexane. The effects of XG and PAM on the interfacial tension follow a similar trend to their effect on surface tension. Particularly, increasing XG concentration leads to a marked elevation in interfacial tension. Compared to the original foam solution, adding 0.10 wt.% XG increases the interfacial tension to 1.25 mN/m with diesel and 1.47 mN/m with cyclohexane, representing increases of 50.60% and 32.43%, respectively. In contrast, PAM concentration exerts a minor effect on the interfacial activity, maintaining interfacial tensions at 0.66 ± 0.03 mN/m with diesel and 1.09 ± 0.05 mN/m with cyclohexane, representing decreases of less than 20%.

CMC-Na exhibits distinctly different effects on the interfacial tension with various oils. With increasing concentration, CMC-Na induces a slight increase in the interfacial tension with diesel, reaching 1.02 mN/m at 0.10 wt.%. Conversely, CMC-Na reduces the interfacial activity between the foam solution and cyclohexane to 1.08 ± 0.03 mN/m, corresponding to a 2.70% reduction. This contrasting phenomenon is primarily attributed to the lower molecular weight of CMC-Na compared to XG and PAM. When in contact with non-polar cyclohexane, CMC-Na preferentially distributes within the bulk solution. This consequently mitigates interference with interfacial aggregation of surfactant molecules, ultimately leading to an improved interfacial activity.

Based on the spreading coefficient results listed in Table 2, a comprehensive analysis was conducted to evaluate the effect of these polymers on the spreadability of foam solutions. The results demonstrate that all foam solutions containing polymers exhibit positive *S_DO_* and *S_CYH_* values, indicating their capability to spread across oil surfaces [39]. Specifically, increasing concentrations of XG and CMC-Na lead to an adverse impact on spreading capability [40]. At a concentration of 0.10 wt.%, the foam solutions containing XG and CMC-Na exhibit reductions in *S_DO_* and *S_CYH_* of 15.78–18.49% and 3.87–6.35%, respectively, compared to the original solution. In contrast, PAM offers a distinct enhancement effect on spreading capacity, as evidenced by a 0.22–4.29% increase in *S_DO_* and *S_CYH_* values.

In conclusion, the influence of these polymeric stabilizers on the interfacial properties of short-chain fluorocarbon surfactant-based foam solutions exhibits significant variation depending on the polymer type. Specifically, both XG and CMC-Na demonstrate competitive adsorption behavior at interfaces, leading to a reduction in interfacial properties and consequent inhibition of foam solution spreading on oil surfaces. In contrast, owing to its amphiphilic structure, PAM enhances surface and interfacial activities, consequently facilitating the spreading capability of foam solutions across oil surfaces.

### 3.2. Effect of Polymeric Stabilizers on the Apparent Viscosity of Foam Solution

**Figure 3:**
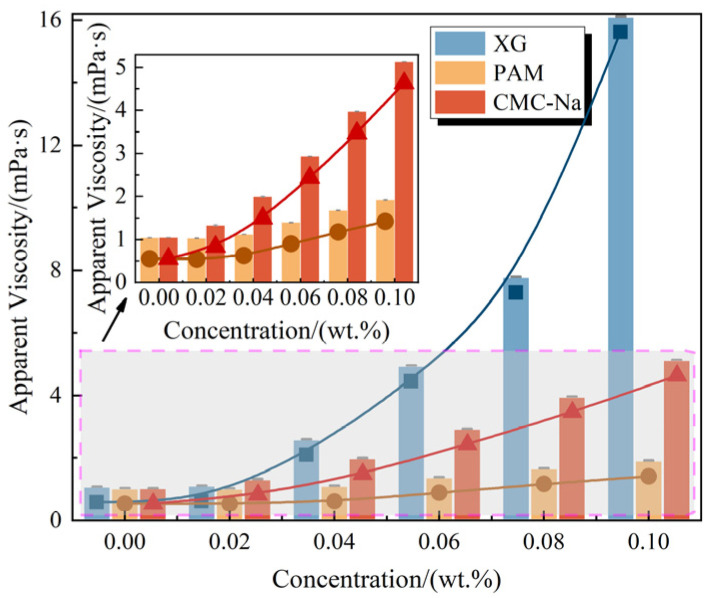
Apparent viscosity (at a fixed rotational speed) of foam solutions containing polymeric stabilizers.

The apparent viscosity (at a fixed rotational speed) of foam solutions containing polymeric stabilizers is illustrated in Figure 3, and the detailed data are provided in Table S1. With increasing polymer concentrations, the apparent viscosity of the foam solution increases significantly. Among these polymeric stabilizers, XG exhibits the most prominent viscosity-increasing effect, CMC-Na ranks second, while PAM possesses the least apparent viscosity enhancement [40]. Specifically, at a concentration of 0.10 wt.%, the solution viscosities are measured at 16.12 mPa·s for XG, 1.92 mPa·s for PAM, and 5.14 mPa·s for CMC-Na, increasing by 1365.45%, 73.64%, and 367.27%, respectively, compared to the original foam solution.

The distinct behaviors of these polymeric stabilizers can be attributed to their molecular structures and interactions with water. As a polysaccharide with double-helical macromolecular structure, XG is rich in hydrophilic functional groups such as –OH and –COO^−^ [41]. This molecular structure facilitates extensive hydrogen bonding with water molecules, constructing a robust three-dimensional hydrogen bond network that effectively restricts the water mobility. Moreover, with increasing XG concentrations, significant intermolecular interaction and chain entanglement occur, leading to an expansion of effective macromolecular dimensions and an elevation of molecular weight. Both of these mechanisms collectively contribute to the substantial increase in apparent viscosity. As a water-soluble cellulose ether, CMC-Na also demonstrates a viscosity-increasing effect by its –COO^−^ functional groups [26]. However, its relatively lower molecular weight and linear structure result in less efficient formation of the hydrogen bond network, leading to a weaker viscosity-increasing effect compared to that of XG [42]. Despite containing hydrophilic amide groups, PAM exhibits limited apparent viscosity enhancement due to its linear structure and relatively weaker hydrophilicity, which constrains the formation of hydrogen bonds with water molecules [43]. These findings collectively demonstrate that the presence of substantial hydrophilic functional groups enables significant alteration of the rheological properties and enhances the apparent viscosity of short-chain fluorocarbon surfactant-based foam solutions through the formation of extensive hydrogen bonds.

However, it is noteworthy that this study is limited to evaluating the apparent viscosity of foam solutions containing polymeric stabilizers. While these results effectively reveal the viscosity-increasing effects of these polymeric stabilizers under a fixed rotational speed, it provides only limited insight. Given that these polymer solutions are non-Newtonian fluids, viscosity measurement conducted at a fixed rotational speed fails to adequately capture the shear-dependent rheological properties. To fully understand the influence of these polymeric stabilizers on the rheological properties of foam solutions formulated with short-chain fluorocarbon surfactant, it is essential to obtain the relationship between solution viscosity and shear-rate (or shear-stress) via specific rheometer.

### 3.3. Effect of Polymeric Stabilizers on the Foam Evolution of Foam Solution

**Figure 4:**
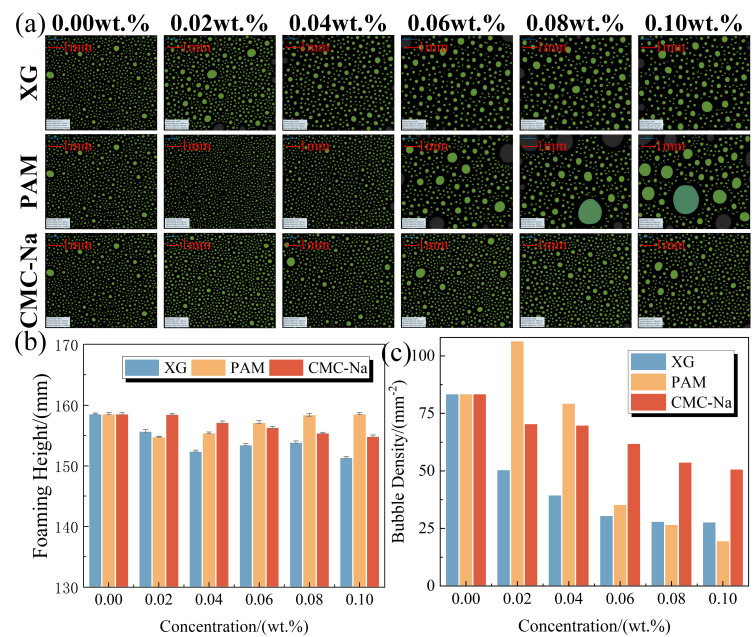
Initial bubble characteristics of foam solutions containing polymeric stabilizers: (**a**) bubble morphology, (**b**) foaming height, and (**c**) bubble density.

The initial bubble morphology, foaming height, and bubble density of foam solutions containing polymeric stabilizers are demonstrated in Figure 4. With the increase in XG and CMC-Na concentrations, the foaming height and bubble density gradually decrease, accompanied by an enlargement of bubble size [28]. Compared to the original foam solution, the initial bubble densities of foam solutions containing 0.10 wt.% XG and 0.10 wt.% CMC-Na are reduced by 66.60% and 40.07%, respectively, indicating a substantial suppression effect on foam formation. This inhibitory phenomenon primarily results from the reduced surface activity and elevated apparent viscosity induced by polymer introduction, which collectively increase the energy required for foam formation and restricts gas diffusion [44,45].

Conversely, PAM exhibits a dual effect on foam formation depending on its concentration. At low concentration of 0.02 wt.%, PAM reduces surface tension, which facilitates the generation of numerous small bubbles and increasing bubble density to 106.59 mm^−2^ [46]. However, this promoting effect diminishes progressively with increasing PAM concentration. In particular, when PAM concentration exceeds 0.06 wt.%, the corresponding foam solutions produce millimeter-scale bubbles with significantly reduced stability. This effect transition is primarily caused by the extensive aggregation of PAM molecules at the bubble film interfaces, along with the formation of entangled network structures. These structures severely impede the uniform distribution of gas during the foaming process, ultimately leading to a marked deterioration in both the quantity and quality of bubbles.

**Figure 5:**
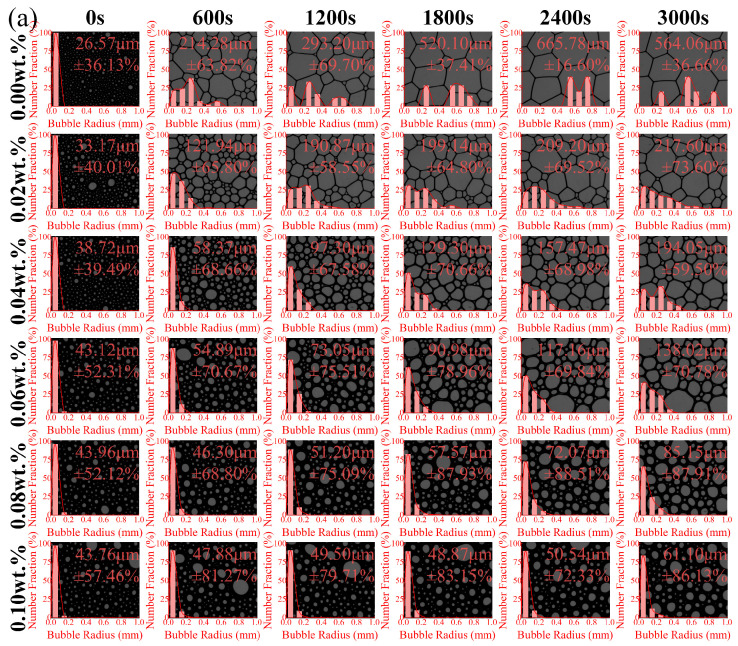

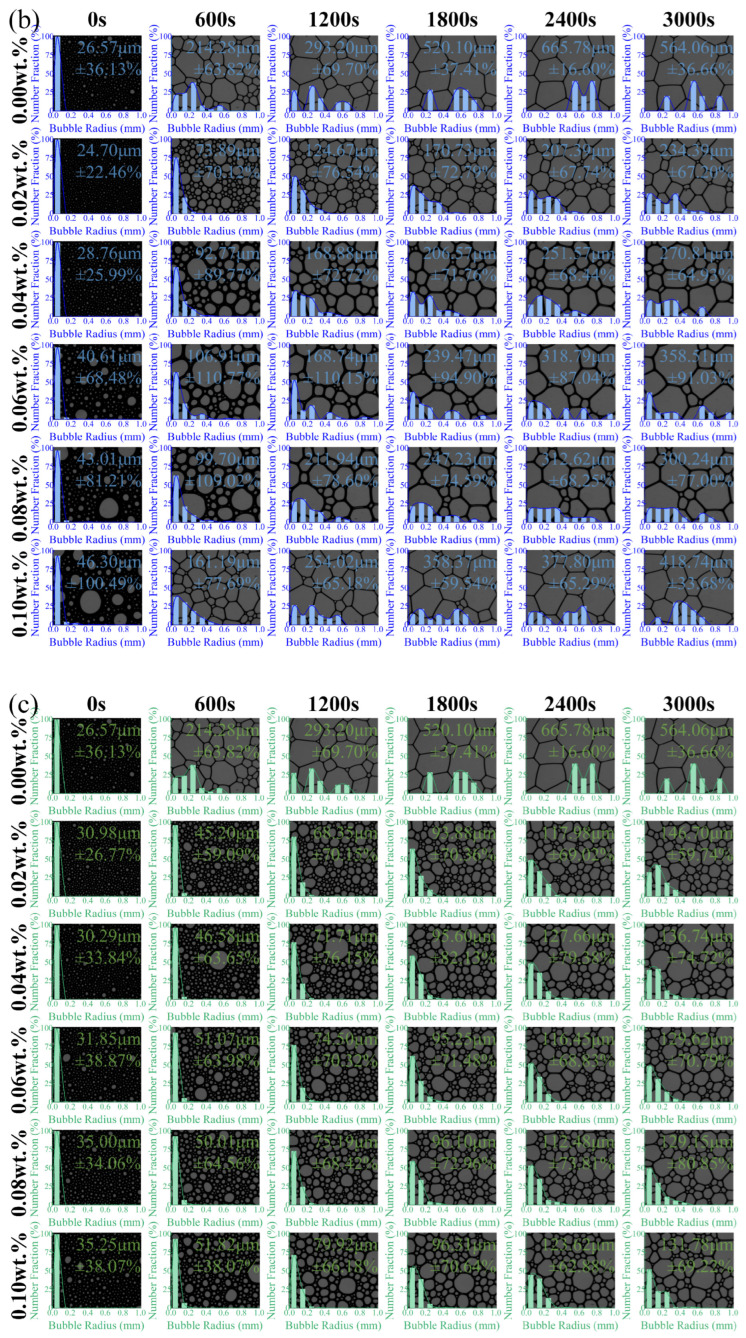
Bubble morphology and size distribution of foam solutions containing polymeric stabilizers at different times: (**a**) XG, (**b**) PAM, and (**c**) CMC-Na.

**Figure 6:**
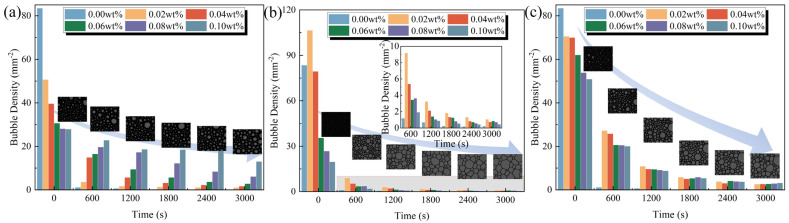
Bubble density of foam solutions containing polymeric stabilizers at different times: (**a**) XG, (**b**) PAM, and (**c**) CMC-Na.

To investigate the influence of polymeric stabilizers on foam evolution, the bubble characteristics of foam solutions containing polymeric stabilizers were analyzed at different times. These critical bubble characteristics, including bubble density, morphology, mean bubble radius (<*r*>), and coefficient of variation (*c_v_*), are presented in Figure 5 and Figure 6. Under the combined effects of gravity drainage, coarsening, and coalescence, the foam undergoes a progressive transformation from small spherical bubbles to larger polyhedral structures, accompanied by a continuous reduction in aqueous film thickness and bubble density [47,48]. By 3000 s, the original foam solution demonstrates a <*r*> value of 564.06 μm and a *c_v_* value of 36.66%, along with a 99.74% decrease in bubble density.

Benefiting from their excellent viscosity-increasing effect, XG and CMC-Na significantly retard foam evolution process. With increasing concentrations of these polymers, the foam solutions demonstrate markedly diminished rates of bubble density decay and size growth. At 3000 s, these bubbles maintain predominantly spherical morphology with a narrow size distribution (≤60 μm). At a concentration of 0.10 wt.%, the final <*r*> values of foam solutions are 10.83–23.36% smaller than that of the original solution, and final bubble densities are maintained at 13.03 mm^−2^ for XG and 3.18 mm^−2^ for CMC-Na.

In contrast, PAM exhibits a limited stabilization effect on bubble evolution. The optimal performance is achieved at 0.02 wt.% PAM, with a 58.45% decrease in final <*r*> value compared to that of the original foam solution. However, this stabilizing effect gradually diminishes with increasing PAM concentration. Particularly, at 0.10 wt.% PAM, the final <*r*> increases to 418.74 μm. This is attributed to the excessive PAM aggregation at the bubble film, which in turn disrupts the surfactant distribution. Furthermore, as a linear polymer, PAM promotes surfactant micellization and forms bridging structures with micelles, consequently compromising the self-recovery capability of bubble films based on the Gibbs-Marangoni effect [49].

**Figure 7:**
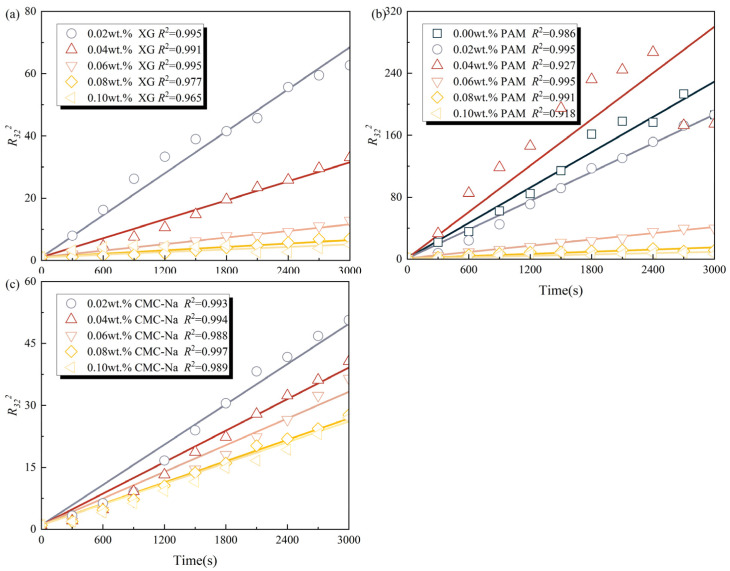
*R*_32_^2^ and fitting curves of foam solutions containing polymeric stabilizers: (**a**) XG, (**b**) PAM, and (**c**) CMC-Na.

As a critical foam destabilization mechanism, foam coarsening is governed by gas diffusion from smaller bubbles to larger ones, driven by the Laplace pressure differences. This process results in the progressive expansion of larger bubbles accompanied by the shrinkage and eventual disappearance of smaller ones. To systematically evaluate the influence of polymeric stabilizers on foam coarsening behavior, the self-similar growth law was employed to assess bubble size evolution [42]. The specific methodology is described in detail in our previous work [16]. The experimental results for the squared dimensionless Sauter radius (*R*_32_^2^) and their fitting curves over time are depicted in Figure 7. The critical parameters, including characteristic coarsening time (*t_c_*), initial mean bubble volume (<*V*_0_>), and effective diffusion coefficient (*D_eff_*) are summarized in Table 3. The fitting results indicate that the self-similar growth law provides excellent agreement (*R*^2^ > 0.95) for bubble size evolution during coarsening of foam solutions containing XG and CMC-Na. However, the solutions containing PAM exhibit lower foam stability and more pronounced bubble size variations during coarsening, resulting in a poorer fitting quality (*R*^2^ > 0.90).

As listed in Table 3, compared to the original foam solution, XG and CMC-Na effectively impede gas diffusion and bubble coarsening, as evidenced by higher *t_c_* value and lower *D_eff_* value. Notably, XG exhibits the most prominent inhibitory effect. For instance, the foam solution containing 0.10 wt.% XG demonstrates a 605.17 s extension in *t_c_* value and a 70.18% reduction in *D_eff_* value compared to equivalent CMC-Na concentrations. These findings strongly support the crucial role of the hydrogen bond network structure offered by XG in retarding bubble coarsening.

PAM demonstrates relatively weaker inhibition of gas diffusion and foam coarsening. The optimal inhibitory effect is attained at 0.02 wt.% PAM, as evidenced by 58.31% reduction in *D_eff_* value and an 18.81% increase in *t_c_* value relative to the original foam solution. However, this inhibitory effect diminishes progressively with increasing PAM concentration. Notably, when PAM concentrations exceeding 0.06 wt.%, the corresponding foam solutions exhibit an abnormal improvement in coarsening characteristics. This anomalous phenomenon does not stem from the actual retardation of foam coarsening, but rather from the significant deterioration in foaming performance at high PAM concentrations. As evidenced in Figure 4, high concentrations of PAM result in larger initial bubble size and broader size distributions. This leads to an increased initial Sauter radius, which consequently reduces the baseline value of the dimensionless Sauter radius (*R*_32_). Under this condition, the fitting results fail to accurately reflect the actual accelerated coarsening phenomenon.

Collectively, although polymeric stabilizers exhibit an inhibitory effect on the foaming performance of short-chain fluorocarbon surfactant-based foam solutions, they demonstrate remarkable efficacy in retarding gas diffusion and bubble coarsening. These effects ultimately enhance foam stability and significantly prolong foam lifetime. However, PAM with an elevated concentration impairs the effective distribution of surfactant molecules at the bubble film interface, consequently compromising bubble structural integrity. This adverse effect subsequently inhibits bubble formation, facilitates gas diffusion, and accelerates foam evolution.

### 3.4. Effect of Polymeric Stabilizers on the Drainage Property of Foam Solution

**Figure 8:**
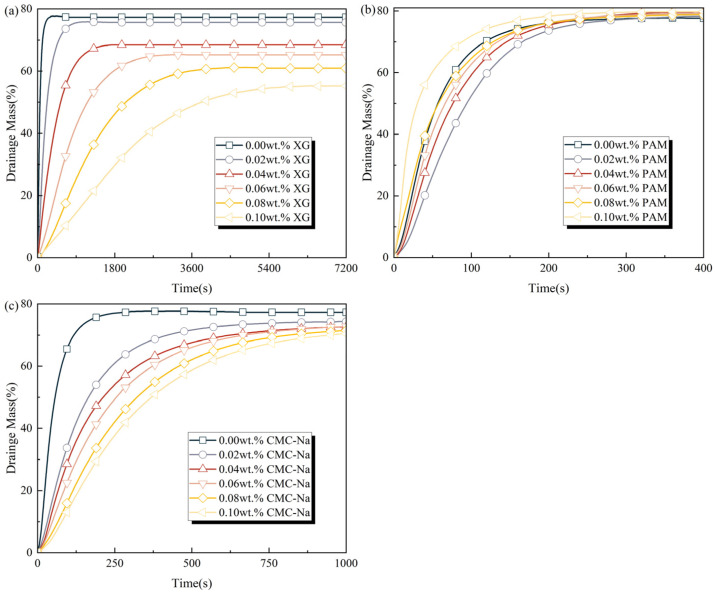
Drainage mass curves of foam solutions containing polymeric stabilizers: (**a**) XG, (**b**) PAM, and (**c**) CMC-Na.

The drainage mass curves of foam solutions containing polymeric stabilizers are illustrated in Figure 8, with the corresponding *t*_25%_ summarized in Table 4. As shown in Figure 8, under the effects of gravity and high temperature, all foams undergo rapid initial drainage, followed by a gradual decrease in drainage rate until reaching equilibrium [42]. XG and CMC-Na exhibit remarkable foam stabilization, significantly retarding the foam drainage process. This enhanced stabilizing effect correlates strongly with increasing concentrations of XG and CMC-Na, where elevated apparent viscosity substantially restricts liquid drainage through foam films. Notably, at 0.10 wt.% XG, the corresponding foam achieves an exceptional *t*_25%_ of 1519.15 s, exhibiting an order-of-magnitude increase over the original foam solution, and maintains the final drained mass below 60%.

PAM exerts a concentration-dependent effect on foam drainage behavior. At concentrations less than 0.06 wt.%, PAM modestly extends *t*_25%_ by 10.08–41.73% compared to the original solution. However, a higher concentration of PAM results in excessive PAM interfacial aggregation and entangling at bubble films, which severely compromises structural stability and accelerates foam drainage. This destabilization becomes particularly pronounced at a PAM concentration of 0.10 wt.%. At this concentration, foam generation is critically inhibited during the ‘double-syringe’ foaming process, resulting in a 57.88% reduction in the *t*_25%_ value compared to the original foam solution.

### 3.5. Mechanism of Polymeric Stabilizers on Foam Solution Performance

**Figure 9:**
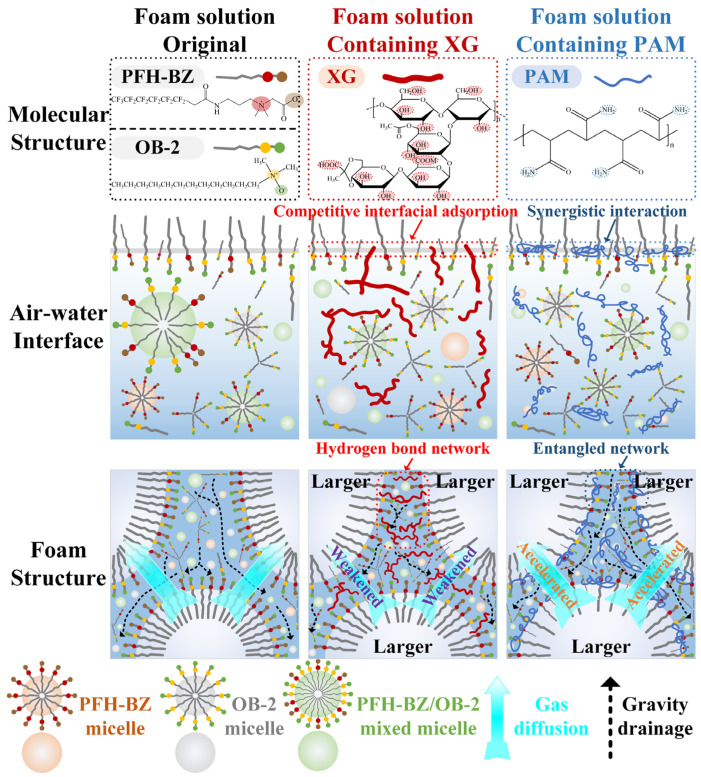
Mechanism schematic of the effect of polymeric stabilizers on foam solution performance.

Based on the above analysis, a possible mechanism elucidating the influence of polymeric stabilizers on short-chain fluorocarbon surfactant-based foams solutions is proposed in Figure 9, selecting XG and PAM as representative polymeric stabilizers. As a polysaccharide polymer rich in hydrophilic groups (–OH and –COOH), XG exhibits minimal interfacial adsorption propensity [37]. Consequently, through competitive adsorption, XG interferes with the ordered distribution of surfactant molecules (PFH-BZ and OB-2) at interfaces, resulting in a marginal decrease in surface activity, interfacial activity, and spreading performance. In contrast, as a linear polymer with pronounced amphiphilic characteristics, PAM enhances the interfacial properties of the short-chain fluorocarbon surfactant-based foam solution.

Regarding foam performance, the hydrophilic groups of XG engage in hydrogen bonding with water molecules, accompanied by increased effective macromolecular dimensions [28,41]. These effects collectively establish an extensive water-holding network, which significantly increases apparent viscosity, thereby inhibiting foam coarsening and drainage. However, PAM demonstrates a limited viscosity-increasing effect. Particularly at high concentrations, PAM aggregates excessively at the bubble films and forms entangled network structures, thus impeding the uniform interfacial distribution of surfactant molecules [49]. This phenomenon compromises bubble film integrity, accelerates foam coarsening and drainage rates, ultimately leading to a deterioration in foaming performance and foam stability.

## 4. Conclusions

In this study, three polymeric stabilizers (XG, PAM, and CMC-Na) were selected to investigate their effects on the interfacial properties, apparent viscosity (at a fixed rotational speed), and foam performance of foam solutions formulated with a zwitterionic short-chain fluorocarbon surfactant (PFH-BZ).

The interfacial properties analysis reveals that XG and CMC-Na exhibit competitive adsorption with surfactant molecules at interfaces. This leads to surface tension increases of 1.44% and 0.50%, respectively, while simultaneously impairing spreading capacity on oil surfaces. In contrast, PAM enhances the interfacial activities of foam solutions, resulting in a 0.30% reduction in surface tension and a 0.22–4.29% increase in spreading capacity across oil surfaces.

The apparent viscosity and foam performance analyses demonstrate that an increasing concentration of XG or CMC-Na significantly elevates apparent viscosity, attributed to the extensive formation of hydrogen bonding networks and the enlargement of effective macromolecular dimensions. Although this apparent viscosity enhancement negatively impacts foaming capacity, it effectively retards foam coarsening and drainage, ultimately improving foam stability. PAM exerts concentration-dependent foam stabilization behavior. At 0.02 wt.%, PAM enhances foam properties, increasing initial bubble density, *t*_25%_ value, and *t_c_* value by 27.59%, 41.73%, and 18.81%, respectively, compared to the original foam solution. However, at higher concentrations, excessive PAM aggregates at interfaces and bridges with surfactant micelles. This critically inhibits surfactant interfacial adsorption and impairs bubble film self-recovery, resulting in substantial deterioration of foam stability. At 0.10 wt.% PAM, the foam solution demonstrates a significant 76.46% reduction in initial bubble density and a 57.88% decrease in *t*_25%_ value compared to the original foam solution.

Comprehensive comparison indicates that XG provides optimal stabilization effect through the formation of hydrogen bond network structures. Specifically, XG minimally affects surface and interfacial activities, while effectively retarding foam coarsening and drainage processes.

This study elucidates the stabilization mechanism of various polymeric stabilizers in short-chain fluorocarbon surfactant systems, providing a theoretical foundation and practical guidance for the development of environmentally friendly and highly efficient foam fire extinguishing agents.

## Figures and Tables

**Table 1:** Components of final foam solutions.

Polymeric Stabilizer (wt.%)	PFH-BZ (mmol/L)	OB-2 (mmol/L)	Urea (wt.%)	Ethylene Glycol (wt.%)	2-(2-Butoxyethoxy)ethanol (wt.%)
0.00	2	4	3	3	1
0.02	2	4	3	3	1
0.04	2	4	3	3	1
0.06	2	4	3	3	1
0.08	2	4	3	3	1
0.10	2	4	3	3	1

**Table 2:** *S_DO_* and *S_CYH_* values of foam solutions containing polymeric stabilizers.

Concentration (wt.%)	*S_DO_* (mN/m)			*S_CYH_* (mN/m)		
	XG	PAM	CMC-Na	XG	PAM	CMC-Na
0.00	5.83	5.83	5.83	4.65	4.65	4.65
0.02	5.46	6.08	5.79	4.39	4.66	4.73
0.04	5.40	6.06	5.66	4.36	4.71	4.64
0.06	5.37	6.05	5.55	4.32	4.73	4.54
0.08	5.23	6.07	5.53	4.15	4.79	4.54
0.10	4.91	6.03	5.46	3.79	4.79	4.47

**Table 3:** <*V*_0_>, *t_c_*, and *D_eff_* values of foam solutions containing polymeric stabilizers.

Concentration (wt.%)	<*V*_0_> × 10^−3^(mm)			*t_c_*(s)			*D_eff_* × 10^−5^/(mm/s)		
	XG	PAM	CMC-Na	XG	PAM	CMC-Na	XG	PAM	CMC-Na
0.00	2.09	2.09	2.09	13.13	13.13	13.13	4.15	4.15	4.15
0.02	3.05	0.73	1.55	44.44	15.60	61.50	1.58	1.73	0.73
0.04	3.73	1.21	1.73	98.33	10.02	78.62	0.82	3.78	0.61
0.06	7.09	10.69	2.29	284.09	158.23	92.85	0.43	1.02	0.62
0.08	7.82	21.95	2.55	543.48	212.31	116.01	0.24	1.23	0.54
0.10	7.25	38.09	2.94	724.64	289.86	119.47	0.17	1.30	0.57

**Table 4:** *t*_25%_ values of foam solutions containing polymeric stabilizers.

Concentration (wt.%)	*t*_25%_(s)		
	XG	PAM	CMC-Na
0.00	30.46 ± 4.13	30.46 ± 4.13	30.46 ± 4.13
0.02	93.37 ± 8.21	43.17 ± 3.07	70.47 ± 3.75
0.04	206.25 ± 8.15	37.25 ± 0.35	85.15 ± 2.65
0.06	505.95 ± 3.45	33.53 ± 1.65	103.30 ± 2.20
0.08	894.50 ± 3.90	27.83 ± 3.49	112.57 ± 7.04
0.10	1519.15 ± 4.15	12.83 ± 1.33	160.53 ± 8.48

## Data Availability

The data presented in this study are available on request from the corresponding author due to privacy.

## Supplementary Materials

The following supporting information can be downloaded at: https://www.mdpi.com/article/10.3390/polym17233112/s1, Table S1: Detailed apparent viscosity (at a fixed rotational speed) data of foam solutions containing polymeric stabilizers.
