# Supplementary material for: Polysaccharides and Polyacrylamide as Linear Polymeric Stabilizers for Zwitterionic Short-Chain Fluorocarbon Surfactant: Interfacial Properties, Apparent Viscosity, and Foam Performance
Source: Polymers (Basel). 2025 Nov 24;17(23):3112. doi: 10.3390/polym17233112 (PMC12693878; doi:10.3390/polym17233112)
Supplement: Supplementary file 1 [file polymers-17-03112-s001.zip › polymers-3982982-supplementary.pdf]

Supporting Information

Polysaccharides and polyacrylamide as linear polymeric stabilizers for zwitterionic short-chain fluorocarbon surfactant: interfacial properties, apparent viscosity, and foam performance

Table S1. Detailed apparent viscosity (at a fixed rotational speed) data of foam solutions containing polymeric stabilizers.

Concentration (wt.%)	Apparent viscosity (mP·s)		
	XG	PAM	CMC-Na
0.00	1.10±0.005	1.05±0.005	1.05±0.004
0.02	1.13±0.005	1.04±0.005	1.33±0.013
0.04	2.60±0.005	1.12±0.005	1.99±0.011
0.06	4.96±0.004	1.40±0.008	2.94±0.005
0.08	7.79±0.008	1.68±0.009	3.97±0.005
0.10	16.12±0.013	1.92±0.010	5.14±0.004
